# Impact of Punicalagin on the Physicochemical and Structural Properties of Wheat Flour Dough

**DOI:** 10.3390/foods8120606

**Published:** 2019-11-22

**Authors:** Hong Peng, Bin Li, Jing Tian

**Affiliations:** 1 College of Food Science and Technology, Huazhong Agricultural University, Wuhan 430070, China; 2 Key Laboratory of Environment Correlative Dietology (Huazhong Agricultural University), Ministry of Education, Wuhan 430070, China

**Keywords:** punicalagin, wheat flour, dough rheology, gluten protein network

## Abstract

The study explored punicalagin (PGN) as a wheat flour enhancer. The impact of PGN on the physicochemical and structural properties of wheat flour have been investigated. It turned out that PGN increased the formation time, stability, tensile resistance, extension, and viscoelasticity of the dough at the concentrations of 0.13 and 0.26 mg/g. Scan electron microscope images of the cross section of the dough displayed a more compact and ordered network structure with the addition of 0.13 and 0.26 mg/g PGN. Fourier transform infrared spectroscopy spectra indicated an increase of α-helix and β-sheet content. However, nonlinear enhancing effects of PGN on the stretching properties, rheology, and structural properties of the dough were observed at concentrations of 0.39 and 0.52 mg/g. Correspondingly, cleavages were observed on the cross section of the dough and the content of β-sheet showed a trend of reduction in the dough with addition of PGN at high concentrations. Taken together, these results indicated the potential usage of PGN as a wheat flour enhancer of natural origin at the concentration below 0.39 mg/g in the flour.

## 1. Introduction

Glutenin is a heterogeneous macromolecular polymer protein that is linked by intermolecular disulfide bonds, which imparts the elasticity and strength of dough [1]. Generally, the elasticity depends on the formation of polymer. Gliadin is a single-chain protein with no interchain disulfide bond or subunit structure. It forms a spherical stereo structure by hydrophobic bonds, hydrogen bonds, and intramolecular disulfide bonds [2], and is responsible for the viscosity of the dough. During dough mixing, gluten proteins are linked together by disulfide bonds, hydrogen bonds, and hydrophobic interactions in the presence of water and mechanical work forming strong crosslinks between and within the polypeptide chains [3,4,5,6]. Wheat dough has multiple usages in the food due to the viscoelasticity, such as bread and noodles [7]. The proper balance between the viscosity and the elasticity/strength of the dough determines the quality of end-products [8]. For instance, too high quantity of gliadin causes a weakening of the protein network with the risk that the produced bread has a wide base and low volume. If the quantity of glutenin prevails, protein network is too rigid with the risk that the produced bread has a reduced volume due to the difficulties of the gas pushing on the gluten network [8]. Therefore, exogenous components are added to improve gluten network and adjust the performance of end products in the volume, texture, and nutrition. These additives include chemical additives, such as iodate [9], ascorbic acid [10] and peroxide [11], enzymes such as glutamyl transferase, glucose oxidase and xylanase [12,13], and microorganisms [14,15]. However, these additives also have certain drawbacks, such as instability, high price, lack of source, or tolerance [11,16]. 

Punicalagin (PGN) is a pomegranate-specific polyphenol that is extracted from pomegranate peel, pomegranate juice, and pomegranate seeds, with the highest content in pomegranate peel [17]. PGN is hydrophilia and stable against heat, ultrasound, ultraviolet radiation, and additives of common food and cosmetics [18]. According to clinical trials, no toxicological treatment-related effects were observed in the human with the oral administration of pomegranate polyphenol extract for up to 2130 mg/day (1305 mg/day of gallic acid equivalents) for 28 days [19] or in the supplementation of a pomegranate extract with trademark of POMx (POM Wonderful, LLC; Los Angeles, CA, USA) in a one-year pilot study [20]. Thus, the consumption of pomegranate extracts including PGN are well tolerated and has been so far safe from a toxicological perspective. Moreover, health benefits were observed including cancer prevention [21], cardiometabolic health promotion [22], and obesity prevention [23] via the supplementation of PGN or pomegranate polyphenol extracts in clinical trials. Therefore, PGN and pomegranate extracts have been highlighted in the field of cosmetics [24], medicine [25], and food [26]. Proprietary pomegranate extracts of POMx and POMELLA (Verdure Sciences, Inc. Noblesville, IN, USA) have been developed as dietary supplementation to improve the fitness [20]. PGN components have been added into cosmetics to lighten the skins via inhibiting melanin [24], promote the regeneration of dermis and epidermis [27], and increase the skin firmness and elasticity [28]. Films containing pomegranate extracts have been applied in food matrix to elongate the shelf life via inhibitory effects on the growth of microorganisms [29,30]. Pomegranate juice has been blended into protein-based film as a plasticizer [31]. 

In recent years, a large number of studies have found that polyphenols enhance the interaction between gluten and improve the rheological properties of the dough [32,33,34,35,36] via alteration of the amount and distribution of disulfide bonds [1,5] to achieve demands in hardness [37,38], moisture [39], and volume [40]. Several studies have reported that the structure and molecular weight of polyphenols play an important role in the interaction of proteins with polyphenols [34]. High molecular weight polyphenols (tannins) have been shown to bind to proteins more strongly or preferentially [41] due to the occupation of more binding sites on proteins. In addition, the flexibility of the polyphenol structure and the number of hydroxyl groups are also indispensable [34]. It has been reported that polymeric procyanidins (sorghum procyanidins) are more effective in enhancing the mixing and stretching properties of the dough via cross-linking than oligomeric procyanidins (grape seed procyanidins) [42]. It is presumed that more hydroxyl groups in polymeric procyanidins occupy more protein binding cites resulting in high potential in the improvement of the dough quality. 

PGN is an exceptional large polyphenol with molecular weight of 1084.7 and with the excellent performance in health benefits as well as in extensive applications. To date, there has not any report of PGN as an additive supplemented to the dough. The presented study aims to investigate the impact of PGN on the physicochemical and structural properties of wheat flour dough, which facilitate the application of pomegranate in the food industry.

## 2. Materials and Methods 

### 2.1. Materials and Chemicals

High-gluten and low-gluten wheat flour were purchased from Zhongyuan Cereals and Oils Co., Ltd. (Henan, China). The protein content of high-gluten and low-gluten wheat flour was 12.2% and 8%, respectively. The wet gluten content of high-gluten flour and low-gluten flour was 31.3% and 27.2% (wt/wt), respectively. Punicalagin (PGN) powder (purity ≥ 98%) separated from pomegranate was purchased from Chengdu Biopurify Phytochemicals Ltd. (Sichuan, China). Other chemicals were purchased from Shanghai Aladdin Biochemical Technology Co., Ltd. (Shanghai, China). Water in the study is ultra-pure with the resistivity of 18.2 MΩ cm.

### 2.2. Sample Preparation

PGN were added to high-gluten flour and low-gluten flour with concentrations of 0.13, 0.26, 0.39, and 0.52 mg/g. Wheat dough was prepared by mixing 5 g of flour and 3.0 mL of water or 5 g of flour with PGN dissolved in 3.0 mL of water with final concentrations mentioned above. Afterwards the mixture was freeze-dried with lyophilizer at −40 °C and 0.1 Pa for 48 h. Then as-prepared dough was ground and screened via a mesh of 180 μm.

### 2.3. Dough Mixing Properties

The dough mixing properties were examined with Brabender Farinograph (Brabender, Duisburg, Germany) according to the previous study [43]. Briefly, 295.5 g of high-gluten wheat flour or 294.5 g of low-gluten wheat flour supplemented with different concentrations of PGN were mixed and stirred at 63 r/min for 1 min in kneading bowls; then, water was added in 25 s to bring the maximum consistency of the dough to approximately 500 FU. Afterwards the recording lasted 20 min. The water absorption rate, formation time, stability, and degree of weakening of the dough were obtained from the recorded curves. The maximum consistency was measured at the development time and in the middle of the curve band width. Each experimental condition contains three replicates of the sample.

### 2.4. Dough Stretching Properties

According to the method of Brabender/International Association for Cereal Science and Technology/Bipea, the dough stretching properties were examined using the Brabender Extenso-graph (Brabender, Duisburg, Germany) as previously reported [43]. Briefly, 150 g of the dough was cut from the dough pre-mixed with Brabender Farinograph for 5 min. Afterwards all the doughs passed through the balling and moulder unit of Brabender Extenso-graph forming uniformly large sticks. As-prepared stick samples were rested in fermentation cabinet for 90 min at 30 °C and tested with the Brabender Extenso-graph. Parameters including the area of the tensile curve, the tensile resistance, and the extension were recorded. At least three replicates of the sample at each condition are prepared for the examination.

### 2.5. Rheological Properties Characterization

Rheological measurements including dynamic oscillatory tests and creep tests were performed using a controlled stress rheometer (Thermo Scientific, Waltham, MA, USA) at 25 °C according to previous studies [13,44]. The dough was prepared as it was described in Section 2.2 and rested for 30 min. Afterwards, the dough was placed into paralleled plates with a diameter of 40 mm and the gap was adjusted to 1 mm. The outer edges of the dough were sealed with glycerin to prevent drying of the sample during the test. A rest time of 5 min was applied for all the samples to allow the relaxation of residual stresses before the measurement. The linear viscoelastic region of the dough was firstly determined via strain sweep procedure in oscillatory mode at the frequency of 1.0 Hz. The dynamic rheological properties of the dough were determined by the frequency sweep at 0.1–10 Hz. Creep and recovery tests were performed via a constant stress (300 s at 100 Pa), followed by a period of no stress (300 s at 0 Pa) according to previous studies [42,45]. A minimum of three replicates of dough samples at each experimental condition were performed.

### 2.6. Observation of Dough Microstructure

The dough containing 2.5% of dry yeast (wt/wt) was slacked for 5 min or fermented for 90 min prior to the flash freezing with liquid nitrogen. Afterwards the frozen dough was immediately crushed and placed in the lyophilizer at −40 °C and 0.1 Pa for 48 h. The surface of the dough section was sprayed with gold particles using an MCI000 ion Sputter (Hitachi, Tokyo, Japan) and the microstructure of the cross section was observed using the scanning electron microscope (SEM) (Hitachi, Tokyo, Japan) at 1000 magnification with a 10.0 kV acceleration voltage.

### 2.7. Measurement of Total and Exposed Free Sulfhydryl (SH) Content

Free and total SH contents of dough samples supplemented with PNG were examined using Ellman’s reagents according to previous studies [46,47]. Two hundred milligrams of dough samples were suspended in 1.0 mL of Tris-gly buffer (0.086 M Tris, 0.09 M glycine, and 0.004 M EDTA, pH 8.0) to quantify exposed sulfhydryl. To quantify total sulfhydryl content, additional 8 M of guanidine hydrochloride was added into the above Tris-gly buffer. Afterwards the suspension was vortexed for 20 min and then centrifuged at 16,000 g for 5 min. Eighty microliter of supernatant was extracted and mixed with the corresponding Tris-gly buffer with or without 8 M of guanidine hydrochloride. Moreover, immediately 40 μL of DTNB working fluid (Tris-gly buffer with 0.01 M 5,5’-Dithio bis-(2-nitrobenzoic acid)) was added into the mixture and incubated at 25 °C for 60 min. The absorbance of the mixture and the reagent buffer as the blank were read at 412 nm. At least three replicates of dough samples at each experimental condition were examined. Free SH content was calculated as follows:

μM SH/g dough = (73.53 × A412 × D)/C(1)

where A412 is the absorbance at 412 nm, D is the dilution factor, C is the sample mass concentration (mg/mL), and 73.53 is derived from 106/(1.36 × 10^4^), where 1.36 × 10^4^ is the molar absorptivity, and 106 is for the calculation from M basis to μM /mL basis and from mg to g [48]. 

### 2.8. Determination of the Content of Free Amino Groups

The change in the content of free amino groups in the dough was determined by the reaction of primary amino groups and o-phthalaldehyde (OPA) according to the previous study [44]. Briefly, 2.0 mL of HCl (0.1 M, pH 1.0) was added into 0.2 g of the dough sample and vortexed for 5 min. Then, the mixture was centrifuged at 9391 g for 10 min. The supernatant (0.1 mL) was mixed with 2.5 mL of OPA reagent. Each 100 mL of OPA reagent contained 50 ml of sodium tetraborate buffer (0.1 M, pH 9.5), 5 mL of 20% sodium dodecyl sulfate solution, 80 mg of OPA dissolved in 2 mL of 95% ethanol, and 0.2 mL of β-mercaptoethanol. The absorbance of the mixture of supernatant and OPA reagent was measured at 340 nm after 2 min of reaction. At least three replicates of dough samples at each experimental condition were determined.

### 2.9. Fourier Transform Infrared Spectroscopy (FT-IR) Characterization

The dough powder sample was ground with potassium bromide at 1/100 ratio. Then, the mixture was pressed into a transparent sheet. Afterwards the air background and the samples were scanned within the range of 400–4000 cm^−1^ with an infrared spectrometer (Thermo Scientific, Waltham, MA, USA) (32 scans at 2 cm^−1^ resolution) at room temperature. At least three spectra of each condition were collected for spectra analyses. The spectra were analyzed with the software Ominc and Peak-Fit 4.12. The peaks at 1600–1700 cm^−1^ were assigned to amide I band; peaks at 1600–1625 and 1625–1640 cm^−1^ were assigned to intermolecular and intramolecular β-sheets [49]; peaks at 1644–1652 cm^−1^ were assigned to random coils; peaks at 1652–1660 cm^-1^ were assigned to α-helices [50]; peaks at 1660–1685 cm^−1^ were assigned to the β-turn [51].

### 2.10. Statistical Analyses

All tests and treatments were expressed as mean ± SD for at least triplicate determinations. The statistical software Origin 8.0 (Microcal Software, Northampton, MA, USA) and IBM SPSS Statistics 25 (SPSS, Chicago, IL, USA) were used for data analyses. The variance (ANOVA) method was applied to analyze significant difference of the obtained data at *p* < 0.05. 

## 3. Results and Discussion

### 3.1. Impacts of PGN on the Mixing Properties of Dough

As expected, the high-gluten dough had a higher mixing characteristics (Table 1) than low-gluten dough including larger water absorption (59.2 versus 58.3%, respectively), longer formation time (1.50 versus 1.47 min, respectively), greater stability time (3.93 versus 3.17 min, respectively) and smaller weakening degree (112.0 versus 120.3 FU, respectively). Apparently, the mixing properties of the dough was related with the gluten content in the flour. 

As it was shown in Table 1, PGN had no significant effect on the water absorption of wheat flour. However, the dough formation time and stability time significantly increased with the addition of PGN. At the same time, the weakening degree decreased. For example, the formation time of high-gluten flour was increased by up to 215% from 1.50 to 4.73 min with the addition of 0.26 mg/g PGN. The formation time of low-gluten flour was increased by up to 24.5% from 1.47 to 1.83 min with the addition of 0.39 mg/g PGN. With the increased addition of PGN, the stability time of the high-gluten dough was increased correspondingly, and it was increased by up to 47.6% from 3.93 to 5.80 min at the concentration of 0.52 mg/g. Meanwhile, the low-gluten flour was increased by up to 30.3% from 3.17 to 4.13 min at the concentration of 0.52 mg/g. The weakening degree of the dough was decreased by up to 32.1% from 112.0 to 76.0 FU prepared with high-gluten flour and by up to 34.6% from 120.3 to 78.67 FU prepared with low-gluten flour in the presence of 0.52 mg/g PGN. The increase in the formation time and stability time indicated an increase in the tolerance of the dough, reflecting the enhancement of PGN on the dough quality. This was likely due to the covalent bonds and hydrogen bonds between PGN and gluten, which increased the strength of the dough [5,52]. However, the enhancement on the low-gluten flour was not as pronounced as that in high-gluten flour, which was likely due to the protein content in the flour. 

### 3.2. Impacts of PGN on the Stretching Properties of Dough

**Figure 1:**
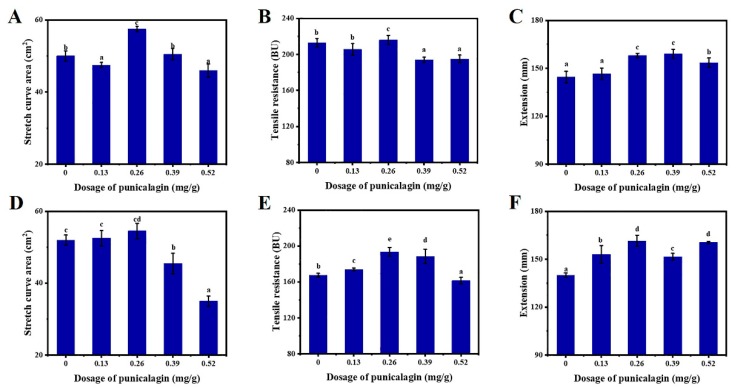
Impact of punicalagin on the tensile properties of the dough (**A**: Stretch curve area of high-gluten dough; **B**: Tensile resistance of high-gluten dough; **C**: Extension of high-gluten dough; **D**: Stretch curve area of low-gluten dough; **E**: Tensile resistance of low-gluten dough; **F**: Extension of low-gluten dough). Different letters above bars indicate significant difference (*p* < 0.05) between treatments at given level.

The stretch curve area (Figure 1A for high-gluten flour and D for low-gluten flour) and tensile resistance of the dough (Figure 1B for high-gluten flour and E for low-gluten flour) were increased with the addition of 0.13 and 0.26 mg/g of PGN. However, the stretch curve area and the tensile resistance of the dough with 0.39 and 0.52 mg/g of PGN were all dropped in both flours. It was assumed that the strong antioxidant properties of PGN at high concentrations (0.39 and 0.52 mg/g) [53] may induce interchanges of sulfhydryl groups and may cause the excessive loss of the bound water in the gluten–PGN system, thereby affecting the secondary structure of the gluten and weakening the dough [51]. 

According to Figure 1, PGN at low concentrations of 0.13 mg/g (in the low-gluten flour dough) and 0.26 mg/g (in the low-gluten and high-gluten flour dough) increased the tensile resistance of the dough while it slightly increased the extension of the dough. This was likely due to dual actions of high molecular weight polyphenols. It strengthened the gluten structure via cross-linking, and stretched the gluten network via breaking disulfide bonds that resulted from antioxidant properties [42]. 

### 3.3. Impacts of PGN on Dough Viscoelasticity and Creep-Recovery Profile

**Figure 2:**
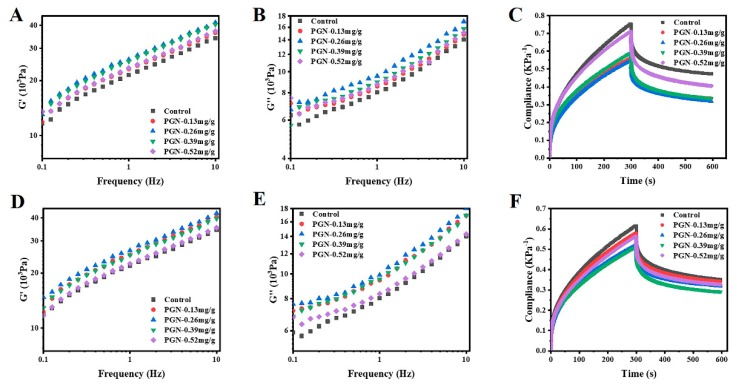
Impact of punicalagin (PGN) on rheology of the dough. (**A**: Elastic modulus G’ of high-gluten dough; **B**: Viscous modulus G’’ of high-gluten dough; **C**: Compliance of high-gluten dough; **D**: Elastic modulus G’ of low-gluten dough; **E**: Viscous modulus G’’ of low-gluten dough; **F**: Compliance of low-gluten dough).

In the program of frequency sweep, for all treatments, G’ (elastic modulus) was higher than G’’ (viscous modulus), tan δ (G’’/G’) < 1 (data not reported), indicating that elasticity was the main feature of the dough, which was in line with recent reports [54]. According to Figure 2 (A and B for high-gluten flour, D and E for low-gluten flour), 0.26 mg/g-PGN-treated dough had the highest G’ and G’’ both in the high-gluten and low-gluten flour. The increase of G’ and G’’ indicated that PGN also increased the viscoelasticity of the dough, which was consistent with the increase of dough tensile properties (Figure 1). The creep and recovery response data curve in Figure 2 (C for high-gluten flour and F for low-gluten flour) showed the typical viscoelastic behavior of the dough. It has been reported that the elasticity of the dough was prominently improved by the addition of proanthocyanins of high molecular weight [42]. In this study, the viscosity and elasticity of the dough were increased at the low concentrations of PGN, which may due to the enhancement of cross-linking in PGN–gluten and protein–protein system [42]. With addition of PGN at high concentrations, the viscoelasticity of the dough was decreased compared to that with addition of PGN at low concentrations due to the reducing potential of PGN in the weakening of the gluten network.

### 3.4. Impacts of PGN on Dough Microstructure

**Figure 3:**
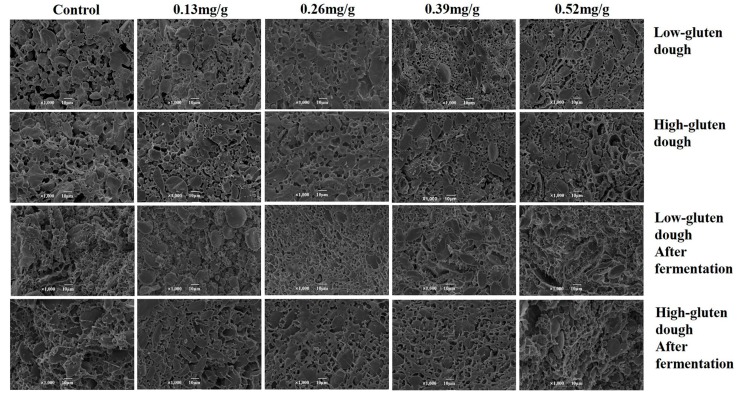
Impact of PGN on the microstructure of the dough cross section examined by scanning electron microscopy.

As it was shown in Figure 3, the dough starch granules without PGN were exposed on the surface and no apparent gluten network structure was observed. With the addition of 0.13 mg/g of PGN, the pores within gluten were less compared to the control group. With the addition of 0.26 mg/g of PGN, a tightly ordered gluten network structure was observed and the starch granules were evenly and orderly distributed in the gluten network structure (Figure 3). Meanwhile, the network structure of the high-gluten flour dough was more compact and well-aligned compared to that of the low-gluten flour dough (Figure 3) with the addition of the same amount of PGN. With the addition of 0.39 and 0.52 mg/g of PGN, significant cleavages were observed in the gluten network structure, which was consistent with the previous results that both tensile resistance and extension of the dough were decreased at the same concentration of PGN. According to SEM of the dough after fermentation, the gluten network structure was partially broken with the addition of 0.39 mg/g of PGN. The breakage was enlarged, and cleavages appeared with the addition of 0.52 mg/g of PGN. These cleavages reduced the gas hold capacity of the dough and directly affected the volume of the end product bread [55]. Taken together, PGN at low concentrations of 0.13 and 0.26 mg/g promoted the formation of gluten network while PGN at high concentrations of 0.39 and 0.52 mg/g caused the formation of cleavages in the gluten network. This was probably because the strengthened cross-linking prevailed at low concentrations and reducing potentials prevailed at high concentrations as well as the dehydration of the gluten network. Moreover, the impacts of PGN on high-gluten flour was more significant than that on low-gluten flour. This was likely due to the cross-linking between PGN and proteins in flours. Higher content of proteins facilitated the formation of a tighter gluten network.

### 3.5. Analyses of Total and Exposed Free Sulfhydryl (SH) and Free Amino Groups Contents

According to Table 2, upon the increase of the amount of PGN, the content of free SH and total SH increased. The increase in sulfhydryl content may due to the strong antioxidant properties of PGN destroying the original disulfide bond between gluten proteins. Both flour type and addition of PGN impacts the interchanges of sulfhydryl groups.

As shown in Table 2, the content of free amino groups was increased in both types of flours with the addition of 0.13 and 0.26 mg/g PGN. Meanwhile it was decreased significantly with the addition of 0.52 mg/g of PGN in high-gluten flour and decreased significantly with 0.39 and 0.52 mg/g of PGN in low-gluten flour. It was assumed that the amount of amino groups was impacted by the release of water via dehydration and the cross-linking with PGN as well as gluten proteins.

### 3.6. Impacts of PGN on Protein Secondary Structure

The texture characteristics of wheat dough mainly depended on the balance of weak and strong physical connections, such as hydrogen bonds, hydrophobic interactions, electrostatic forces, covalent bonds, and disulfide bonds [56]. 

As the addition of PGN was increased, α-helix, β-sheet, and random coil were increased, while β-turn was decreased (Table 3), suggesting β-turn was converted to α-helix, β-sheet, and random coil. In general, β-sheets were considered to be the most stable secondary structure, and an increase in the formation of α-helix would result in a more ordered structure [57]. As shown in Table 3, the structure of β-sheets and α-helix increased with the addition of PGN, indicating a more stable and well-aligned gluten secondary structure. This result was consistent with previous data that PGN at low concentrations enhanced the gluten network structure and improved dough rheological properties. However, the stability of the structure was not linearly correlated with the amount of PGN addition. This was likely correlated with the moderate dehydration of gluten caused by PGN at low concentration, which promoted the conversion of β-turn to β-sheet and stabilized the network structure. At high concentrations the reducing potential of PGN prevailed and may cause the decrease of β-sheet content [58]. Moreover the application of high amount of PGN may release water from hydrogen-bonded cluster and cause the dehydration of dough, thus weakening the gluten mesh [51].

## 4. Conclusions

In the present study, PGN increased the formation time of high-gluten flour dough by up to 215% and the low-gluten flour dough by up to 24.5%. It increased the stability time of the dough prepared with high-gluten flour maximally by 47.6% and that with the low-gluten flour maximally by 30.3%. PGN at low concentrations of 0.13 and 0.26 mg/g increased the tensile resistance, extension capacity, and rheological properties of the dough. Among them, the most prominent increase was observed for the dough with 0.26 mg/g of PGN, where stretching resistances were increased by 1.4% and 15.5% for the high- and low-gluten flour dough respectively; extensions were increased by 9.3% and 15.4% for the high-gluten and low-gluten flour dough, respectively. The morphology of cross section of the dough appeared a more compact and ordered network structure in the presence of PGN at low concentrations. These characteristics can be explained by the increased content of α-helix and β-sheet possibly via the water redistribution and cross-linking in PGN-gluten system. These nonlinear correlation of PGN addition and dough quality was unlike most polyphenols or traditional gluten strengthening agents. Too high quantity of PGN in the dough may physically disrupt the gas cells and gluten network producing poor quality of bread and noodles. Taken together these results indicated that PGN can be used as a flour enhancer of natural origin. The suitable concentrations for the application of PGN in the dough were around 0.26 and below 0.39 mg/g. The enhancing effects of PGN were more prominent in the high-gluten flour.

## Figures and Tables

**Table 1:** Impact of punicalagin on the mixing properties of the dough.

Flour	Add Amount (mg/g)	Water Absorption Rate (%)	Formation Time (min)	Stability (min)	Weakening Degree (FU)
High-gluten	0	59.2 ± 0.10 ^a^	1.50 ± 0.01 ^a^	3.93 ± 0.15 ^a^	112.0 ± 4.58 ^a^
	0.13	59.3 ± 0.25 ^a^	1.83 ± 0.11 ^a^	4.57 ± 0.72 ^a^	91.7 ± 14.6 ^bc^
	0.26	59.3 ± 0.10 ^a^	4.73 ± 1.05 ^b^	5.57 ± 0.15 ^b^	92.7 ± 7.37 ^b^
	0.39	59.3 ± 0.06 ^a^	4.60 ± 0.44 ^b^	5.80 ± 0.20 ^b^	76.7 ± 4.93 ^bc^
	0.52	59.3 ± 0.06 ^a^	4.73 ± 0.42 ^b^	5.80 ± 0.36 ^b^	76.0 ± 7.21 ^c^
Low-gluten	0	58.3 ± 0.20 ^a^	1.47 ± 0.06 ^a^	3.17 ± 0.32 ^a^	120.3 ± 11 ^a^
	0.13	58.3 ± 0.06 ^a^	1.63 ± 0.15 ^ab^	3.30 ± 0.10 ^b^	103.7 ± 17 ^a^
	0.26	58.2 ± 0.06 ^a^	1.70 ± 0.20 ^ab^	3.37 ± 0.21 ^bc^	118.3 ± 4.5 ^a^
	0.39	58.3 ± 0.06 ^a^	1.83 ± 0.35 ^c^	3.83 ± 0.25 ^cd^	103.3 ± 13 ^a^
	0.52	58.2 ± 0.10 ^a^	1.57 ± 0.15 ^a^	4.13 ± 0.38 ^d^	78.67 ± 5.5 ^b^

Values followed by the same letter in the same column (within the same flour) are not significantly different (*p* < 0.05). Two-ways ANOVA analysis: Water absorption rate: F_Flour_ = 520.20, *p* < 0.05, F_Addition_ = 0.24, *p* > 0.05, F_interaction_ = 0.53, *p*_interaction_ > 0.05; Formation time: F_Flour_ = 149.95, *p* < 0.05, F_Addition_ = 27.05, *p* < 0.05, F_interaction_ = 22.14, *p*_interaction_ < 0.05; Stability time: F_Flour_ = 168.27, *p* < 0.05, F_Addition_ = 19.38, *p* < 0.05, F_interaction_ = 4.42, *p*_interaction_ < 0.05; Weakening degree: F_Flour_ = 17.03, *p* < 0.05, F_Addition_ = 10.60, *p* < 0.05, F_interaction_ = 4.25, *p*_interaction_ < 0.05.

**Table 2:** Impact of PGN on the content of sulfhydryl groups (SH) and free amino groups in dough.

Flour	Add Amount (mg/g)	Free SH (μM/g dough)	Total SH (μM/g dough)	Free Amino Groups Absorbance
High-gluten	0	0.38 ± 0.005 ^a^	0.57 ± 0.012 ^a^	0.051 ± 0.008 ^b^
	0.13	0.39 ± 0.005 ^a^	0.62 ± 0.014 ^b^	0.072 ± 0.001 ^c^
	0.26	0.41 ± 0.003 ^bc^	0.64 ± 0.004 ^bc^	0.070 ± 0.012 ^c^
	0.39	0.43 ± 0.013 ^c^	0.66 ± 0.005 ^c^	0.069 ± 0.007 ^c^
	0.52	0.40 ± 0.002 ^ab^	0.77 ± 0.018 ^d^	0.035 ± 0.008 ^a^
Low-gluten	0	0.38 ± 0.007 ^a^	0.56 ± 0.006 ^a^	0.047 ± 0.005 ^a^
	0.13	0.38 ± 0.004 ^ab^	0.60 ± 0.002 ^a^	0.071 ± 0.006 ^b^
	0.26	0.39 ± 0.001 ^bc^	0.60 ± 0.005 ^a^	0.072 ± 0.017 ^b^
	0.39	0.39 ± 0.002 ^bc^	0.71 ± 0.035 ^b^	0.050 ± 0.011 ^a^
	0.52	0.40 ± 0.009 ^c^	0.71 ± 0.024 ^b^	0.047 ± 0.015 ^a^

Values followed by the same letter in the same column (within the same flour) are not significantly different (*p* < 0.05). Two-ways ANOVA analysis: Free SH: F_Flour_ = 33.71, *p* < 0.05, F_Addition_ = 16.17, *p* < 0.05, F_interaction_ = 6.34, *p*_interaction_ < 0.05; Total SH: F_Flour_ = 5.34, *p* < 0.05, F_Addition_ = 71.89, *p* < 0.05, F_interaction_ = 6.23, *p*_interaction_ < 0.05; free amino groups: F_Flour_ = 1050.95, *p* < 0.05, F_Addition_ = 1042.49, *p* < 0.05, F_interaction_ = 417.25, *p*_interaction_ < 0.05.

**Table 3:** Impact of PGN on the secondary structure of dough proteins.

Flour	Add Amount (mg/g)	α-helix (%)	β-sheet (%)	β-turn (%)	Random Coil (%)
High-gluten	0	14.98 ± 0.05 ^a^	29.90 ± 0.04 ^a^	40.70 ± 0.07 ^a^	14.43 ± 0.01 ^a^
	0.13	15.83 ± 0.01 ^d^	31.84 ± 0.05 ^b^	37.78 ± 0.06 ^b^	14.56 ± 0.05 ^b^
	0.26	15.52 ± 0.14 ^bc^	32.35 ± 0.12 ^d^	37.11 ± 0.16 ^d^	15.03 ± 0.12 ^d^
	0.39	15.64 ± 0.04 ^c^	32.04 ± 0.08 ^c^	37.58 ± 0.08 ^c^	14.74 ± 0.02 ^c^
	0.52	15.41 ± 0.02 ^b^	32.27 ± 0.09 ^d^	37.43 ± 0.08 ^c^	14.88 ± 0.05 ^d^
Low-gluten	0	14.81 ± 0.16 ^a^	29.21 ± 0.17 ^a^	41.89 ± 0.15 ^a^	14.10 ± 0.13 ^a^
	0.13	15.56 ± 0.07 ^c^	32.73 ± 0.05 ^e^	36.90 ± 0.09 ^d^	14.81 ± 0.04 ^b^
	0.26	15.38 ± 0.06 ^b^	32.26 ± 0.05 ^d^	37.33 ± 0.03 ^c^	15.04 ± 0.03 ^c^
	0.39	15.56 ± 0.07 ^c^	31.91 ± 0.05 ^c^	37.75 ± 0.02 ^b^	14.78 ± 0.06 ^b^
	0.52	15.69 ± 0.05 ^d^	31.37 ± 0.08 ^b^	37.76 ± 0.03 ^b^	15.18 ± 0.04 ^d^

Values followed by the same letter in the same column (within the same flour) are not significantly different (*p* < 0.05). Two-ways ANOVA analysis: α-helix: F_Flour_ = 4.11, *p* > 0.05, F_Addition_ = 56.68, *p* < 0.05, F_interaction_ = 6.31, *p*_interaction_ < 0.05; β-sheet: F_Flour_ = 17.96, *p* < 0.05, F_Addition_ = 609.39, *p* < 0.05, F_interaction_ = 56.26, *p*_interaction_ < 0.05; β-turn: F_Flour_ = 28.16, *p* < 0.05, F_Addition_ = 1595.06, *p* < 0.05, F_interaction_ = 72.45, *p*_interaction_ < 0.05; random coil: F_Flour_ = 2.17, *p* > 0.05, F_Addition_ = 75.98, *p* < 0.05, F_interaction_ = 11.59, *p*_interaction_ < 0.05.
